# Dose distribution changes with shielding disc misalignments and wrong orientations in breast IOERT: a Monte Carlo – GEANT4 and experimental study

**DOI:** 10.1120/jacmp.v13i5.3817

**Published:** 2012-09-06

**Authors:** Giorgio Russo, Carlo Casarino, Gaetano Arnetta, Giuliana Candiano, Alessandro Stefano, Filippo Alongi, Giovanni Borasi, Cristina Messa, Maria C. Gilardi

**Affiliations:** ^1^ Institute for Molecular Bio‐imaging and Physiology (IBFM) National Research Council (CNR) Segrate Milano; ^2^ Fondazione Istituto San Raffaele G. Giglio Cefalù Palermo; ^3^ Laboratorio di Tecnologie Oncologiche (LATO) Cefalu' Palermo; ^4^ University of Milano‐Bicocca Milano; ^5^ Department of Radiation Therapy San Raffaele Scientific Institute Milano; ^6^ San Gerardo Hospital Monza Italy

**Keywords:** breast intraoperative radiotherapy, shielding disc misalignment, dose distribution, Monte Carlo Geant4 simulation

## Abstract

One of the most relevant risks in breast intraoperative electron radiotherapy (IOERT) is the incorrect positioning of the shielding disc. If such a setup error occurs, the treatment zone could receive a nonuniform dose delivery, and a considerable part of the electron beam could hit — and irradiate — the patient's healthy tissue. However misalignment and tilt angle of the shielding disc can be evaluated, but it is not possible to measure the corresponding *in vivo* dose distribution. This led us to develop a simulation using the Geant4 Monte Carlo toolkit to study the effects of disc configuration on dose distribution. Some parameters were investigated: the shielding factor (SF), the radiation back scattering factor (BSF), the volume–dose histogram in the treatment zone, and the maximum leakage dose (MLD) in normal tissue. A lateral shift of the disc (in the plane perpendicular to the beam axis) causes a decrease in SF (from 4% for a misalignment of 5 mm to 40% for a misalignment of 40 mm), but no relevant dose variations were found for a tilt angle until 10°. In the same uncorrected disc positions, the BSF shows no significant change. MLD rises to 3.45 Gy for a 14 mm misalignment and 4.60 Gy for 30° tilt angle when the prescribed dose is 21 Gy. The simulation results are compared with the experimental ones, and allow an *a posteriori* estimation of the dose distribution in the breast target and underlying healthy tissue. This information could help the surgical team choose a more correct clinical setup, and assist in quantifying the degree of success or failure of an IOERT breast treatment.

PACS number: 87.53.Jw, 87.55.dk, 87.55.Gh, 87.55.K‐, 87.55.N‐

## I. INTRODUCTION

Intraoperative electron radiotherapy (IOERT) is a radiotherapy technique that delivers a single dose of radiation directly to the tumor bed, or to the exposed tumor, during surgery. It is mainly used as an adjuvant to surgery or as a preliminary boost to be followed later by fractionated conventional external whole‐breast radiotherapy (WBRT).^(1)^ The objective is to achieve a higher dose in the target volume, while dose‐limiting structures are surgically displaced.^(2)^

For this purpose, a new generation of mobile linear accelerators like NOVAC7 (NRT, Aprilia, Italy),^(3)^ Liac (Sordina SpA, Italy),^(4)^ and Mobetron (IntraOp Medical, Inc. Santa Clara, CA)^(5)^ have been designed to deliver radiation therapy in the operating theater. A NOVAC7 system has been installed at our hospital to be used specifically for breast cancer treatment.

The main rationale of IOERT treatment is to improve patients' life quality. IOERT is very appropriate for the new trends in breast cancer management where mastectomy is substituted by a more conservative treatment, more appropriate for limited‐stage breast tumors.^(2)^

The development and current availability of an intraoperative radiotherapy technique to be used directly during the surgery session has increased the need for reconsidering previous consolidated treatment procedures and planning new clinical protocols. Indeed, the choice of an intraoperatory radiotherapy treatment, and its application procedure, depends on the nature of the tumor and on the specific patient anatomical–pathological situation. In this scenario, it is of fundamental importance to plan the best clinical setup that optimizes the dose distribution in each treatment procedure, but also one that, at the same time, takes great care that the patient's healthy tissue adjacent to the treatment volume is protected.

To date, for IOERT, there is no Monte Carlo treatment planning system (TPS) available that can accurately study dose distribution near inhomogeneous tissue, nor are there controlled and automated delivering dose systems. The process leading to an IOERT treatment is mostly the result of a sequence of manually handled actions involving the surgeon, medical assistants, radiotherapist, medical physicists, and technicians

In this context, one of the subprocesses during breast cancer IOERT treatment is the protection of internal normal tissue from radiation leakage.^6^, ^7^ It involves the surgeon positioning a metal disc between the deep face of the patient's residual breast and the pectoral muscle, and suturing the resected mammary gland. Measurements of target volume thickness and applicator placement follow.

In high‐dose IOERT, the use of a shielding disc is mandatory.^(6)^ Tumor tissue must be irradiated uniformly, almost to within 90% of the maximum prescribed dose (23Gy). For depths of 10–25 mm, the real dose distribution in a IOERT treatment can result, depending on the beam energy used, in such uniformity characteristics. Nevertheless, for deeper zones, the distribution can be seen to be a descending sigmoid profile. Thus, without the disc the residual dose involves normal tissues.

A two‐layered disc configuration is frequently utilized. The first layer (made of PMMA, aluminum) has two functions: to slow down the beam electrons and to adsorb the back scattered radiation produced by the second layer. The second layer, made of higher density and atomic number material (lead, copper, etc.) stops the residual electrons.^8^, ^9^, ^10^

In clinical setup, the shielding disc diameter is greater than the diameter of the internal applicator, a condition that may avoid dose leakage into normal tissue.

As Ciocca et al.^(11)^ have shown, the execution of internal normal tissue protection and applicator placement processes are strictly related to two very relevant risks in IOERT treatment: misalignment and wrong orientation of the shielding disc (this latter for a two‐layered disc).

The first risk is the incorrect positioning of the shielding disc with respect to the applicator placement and vice versa. Such an error, subject to the lack of direct visual disc control, can lead to the treatment zone receiving a nonuniform dose delivery, as well as irradiation to normal tissues like the pectoralis muscle, the ribs, and underlying lung tissue.

The second risk is error in disc placement, often affected by the fact that the disc is encapsulated in a (sterilized) light opaque package. This error causes incorrect overdose delivery and dose distribution in the treatment region.

At present, there is no way to exactly control the correctness of the disc position *in vivo* and thus avoid the above potential errors. Nevertheless, the position can be partially checked during the measurement of the target volume thickness, and also *a posteriori*, by analyzing the GAFCHROMIC film at the front of the disc applied during surgery. However, these analyses give no information about the dose distribution in the treatment region and underlying normal tissue.

Our study aimed to quantify, by Monte Carlo simulation, how the dose distribution changes when disc misalignment and/or wrong orientation occur. In this way, given the disc position known by *in vivo* and *a posteriori* measurements, such simulations would provide a more realistic dose distribution in the tissue treatment region and the hot spots that occur in healthy tissue during treatment. These results could assist the surgeon and radiotherapist in choosing the clinical setup, allowing them to make more informed and correct decisions, and would, furthermore, lead to the quantification of the degree of success or failure of an IOERT breast treatment and a better evaluation of prospective approaches for risk assessment.^(11)^

## II. MATERIALS AND METHODS

### A. Experimental and simulated setup

NOVAC7 (NRT, Aprilia, Italy) is an IOERT system producing electron beams of 4, 6, 8, and 10 MeV nominal energies to perform treatments at different tissue depths. Applicators with different diameters from 3 to 10 cm and slant angles of 0°, 15°, 22.5°, 30°, and 45° are available.

(Figure 1a) illustrates the NOVAC7 in the surgery room; (Fig. 1b) shows the final part of the NOVAC7. The first block constitutes the accelerator head, within which is the titanium exit window and a cylindrical PMMA structure where two monitor chambers are installed. The accelerator head acts as the primary collimator system, while the secondary collimation obtained by a larger PMMA cylinder divides into two parts through a fixed adaptor and a final collimator of variable length, diameter, and slant angle. A PTW MP3 water phantom (PTW, Freiburg, Germany) (box of 200 mm×200 mm×200 mm) is positioned at the end of the collimator.

**Figure 1(a):**
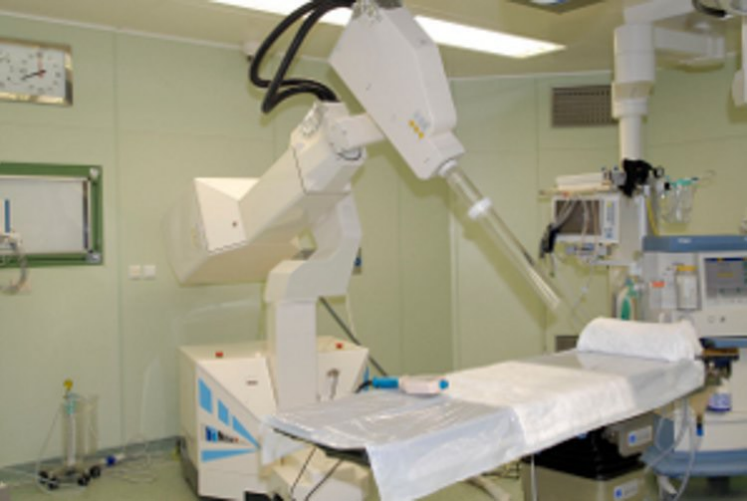
The mobile linear electron accelerator NOVAC7 in the operating theatre.

(Figure 1c) also shows the shielding disc. It is double‐layered, the first layer being aluminum 4 mm in thickness, while the second is comprised of 2 mm thick lead. The disc diameter is 12 cm, 2 cm more than the secondary collimator.

Experimental percentage profile dose (PPD) parameters (field size, symmetry, homogeneity) and percentage depth dose (PDD) reference points (R100, R90, R50, R30 and R10, where Rx is the depth corresponding to x percentage of the maximum dose released) were measured in reference conditions: source‐to‐phantom surface distance, SSD=100 cm, 100 mm collimator diameter, and 0° angle.^(12)^ The experimental data were acquired inside the water phantom using a p‐type silicon diode detector (sensitive area $2\;\times \;2\;{\text{mm}}^{2}$). The profile measurements were obtained by moving the detector along the z‐ or y‐axis at R100 PDD depth by steps of 2.0 mm for the first 40 mm, of 4.0 mm between 40 and 100 mm, and 2.0 mm until 140 mm. The PDD measurements were obtained moving the detector along the beam axis (x‐axis) by steps of 0.5 mm for the first 24 mm, of 1.0 mm between 24 and 34 mm, and 2.0 mm until 70 mm.

The simulations were performed with the Geant4 toolkit. This package allows the simulation of the passage and interaction of particles with matter. It is written in C++ language code and based on Monte Carlo methods. Originally developed to describe nuclear processes in high‐energy accelerators, Geant4 was subsequently revised to include interactions at lower energy (down to 250 eV). Thus, today, it is widely adopted by the Medical Physics community.^13^, ^14^

The toolkit offers the user a flexible structure to model the geometry, materials, primary particles and management of events. Different implementations of physics processes are used, providing equivalent or alternative modeling approaches. Indeed, for electrons, positrons, and photons, different physics lists are available,^(15)^ all of them taking into account: photoelectric effect, Compton and Rayleigh scattering and gamma conversion for photons; multiple‐ scattering, ionizations and Bremsstrahlung (Annihilation) for electrons (for positrons). The main differences are the physical models used and, consequently, the covered energetic range. For example, the Em_option3 physics list covers the above physics processes for electron energies in the range 1 keV–10 GeV. The LowEm_Penelope (the Geant4 implementation of the physics models developed for the PENELOPE code^(16)^) in the range from few keV up to about 1 GeV substitutes, in part, the physics models utilized in the corresponding Em_option3 physics list. The LowEm_Livermore physic list operates in a similar way, but it makes direct use of atomic shell cross‐sectional data to calculate the low energy processes. From the above, we selected the LowEm_Penelope because the PENELOPE model has been expressly implemented for Monte Carlo simulation and for low‐energy electron–positron processes.^16^, ^17^

For our purposes, we developed a Monte Carlo application program, *iort_therapy*, published in the advanced examples of the official Geant4 release (9.5 version). *Iort_therapy* was implemented to address typical needs related to the intraoperative radiotherapy (IORT) technique. Such needs can include the calculation of dose distribution curves in water or other materials, the possibility of choosing from among different clinical setups, and the study of radioprotection devices. Via macro file, the user can easily select the appropriate collimator beam line system, the phantom and detector dimensions, the initial conditions of the electron beam (position and momentum distributions), and the appropriate physics list.

**Figure 2:**
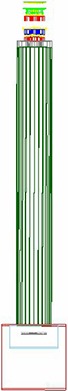
Simulated collimation system of the Novac7 as output from *iort_therapy* application. Upper: content of outer side of the accelerator head — titanium exit window and cylindrical PMMA structures. Middle: secondary collimation system (larger PMMA cylinder). Lower: water phantom (red box); inside it, the sensitive detector (blue box) and the shielding disc (gray).

Figure 2 shows the simulated geometric system configuration (from the titanium exit window down to the water phantom) as output from *iort_therapy*. The shapes, dimensions, and materials were adapted according to technical specifications made available by the NOVAC7 manufacturer.^(3)^ The upper part represents the outer side of the accelerator head — the titanium exit window and the cylindrical PMMA structures. The central part is the secondary collimation system (larger PMMA cylinder) and at the end, the water phantom, represented by the red box. Inside is the sensitive detector (blue box 70 mm depth×150 mm×150 mm surface) positioned where the shielding disc (120 mm diameter) can be inserted (also shown). The $150\times 150\;{\text{mm}}^{2}$ sensitive area allows us to study the dose distribution in the whole treatment and normal tissue zones (see also Fig. 4). Of particular interest is the region around the shielding disc where the greatest amount of diffused radiation is generated. To compare the simulation data with the experimental, the sensitive detector area was restricted so as to correspond to the p–type silicon diode sensitive detector area ($2\;\times \;2\;{\text{mm}}^{2}$).

**Figure 1(b):**
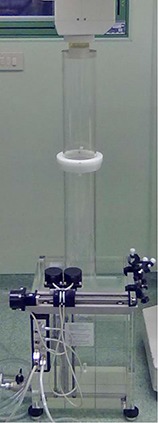
Experimental setup: NOVAC7 beam collimation system. Upper: accelerator head, inside (not visible) is a titanium exit window, a cylindrical PMMA structure, and two monitor chambers. Middle: PMMA cylinder with fixed adaptor and final collimator. Lower: A PTW MP3 water phantom (box of 200 mm×200 mm×200 mm).

The sensitive detector volume was subdivided in voxel of $0.5\;\times \;0.5\;\times \;0.5\;{\text{mm}}^{3}$, and the cut in range for electrons was set to 0.01 mm, corresponding to follow particles with energy up to 25 keV in water and 12 keV in lead. In this way, the simulation has better resolution than experimental measurements.

### B. Monte Carlo validation

The comparison between the simulated and the experimental PPDs and PDDs in water phantom was made in the reference conditions and without the shielding disc. To reproduce the active surface of a p‐type silicon diode, the sensitive area of the detector was restricted to $2\;\times \;2\;{\text{mm}}^{2}$. To provide a relative error comparable to that obtained with the p‐type silicon diode apparatus (0.3%), $160\times 10^{6}$ histories were generated per simulation.

The physical characteristics of the electron beam (energy, spatial, and moment distributions) at the source (i.e., at the level of the titanium exit window (see Fig. 2), are assumed to be represented by Gaussian functions.^18^, ^19^ The respective mean values and variances were iteratively adjusted to achieve, between simulated and experimental PPD and PDD curves, the best match (i.e., a root square mean relative difference within 2% for the PDD reference values, within 3% and 5% for profile symmetry and homogeneity parameters, respectively). The mean energy, ${\overset{ˉ}{E}}_{0}$, and the most probable beam energy, $E_{0p}$, at the water phantom surface were also calculated according to, respectively, the IAEA TRS 398 and the AAPM reports 32.^12^, ^20^

### C. Simulated clinical setup

To study the dose distributions in the treatment region and in the underlying normal tissue, PDD curves were calculated considering the entire sensitive area of the detector ($150\;\times \;150\;{\text{mm}}^{2}$).

The dose distributions were calculated with the disk in both the correct position, ((Fig. 3a) and some erroneous configurations, like parallel misalignment (from 5.0 mm to 42.0 mm) and rotations (from 2.5° to 30.0°), as shown in (Fig. 3b) and (c).

**Figure 1(c):**
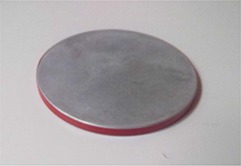
Shielding disc (double‐layered): the first being aluminum 4 mm thickness, the second comprised of 2 mm thick lead. Disc diameter is 120 mm.

In addition, we considered the extreme condition (i.e., when the disc is 180° rotated (wrong orientation error)), thus presenting the lead face to the impinging beam.

To reproduce the possible treatment configurations, the disc was placed at the R90 depths (10.5 mm for 4 MeV, 14.5 mm for 6 MeV, 20.0 mm for 8 MeV, and 24.5 mm for 10 MeV) and in three intermediate depths (12.5 mm for 6 MeV, 16.8 mm for 8 MeV, 22.8 mm for 10 MeV).

To analyze and quantify the effects on dose distribution due to a particular disc configuration, three parameters were estimated: the shielding factor (SF), the backscattering factor (BSF), and the maximum leakage dose (MLD).

The SF specifies the shielding level in normal tissue after the disc. It is defined as $100*(1-D_{d}/D_{\text{nd}})$, where $D_{\text{nd}}$ is the dose released in the normal tissue (gray area in Fig. 4) in the absence of the disc, while $D_{\text{nd}}$ is the analogous quantity but in the presence of the disc (dark gray area in Fig. 4). With this definition, SF is an a‐dimensional factor that takes into account both the physical–geometrical properties of the disc (material, shape, position, orientation) and the initial characteristics of the beam.

The BSF quantifies the dose of backscattered radiation from the disc into the breast, and is defined as the ratio of PDD values with and without disc at the same depth in the treatment region.^8^, ^9^

MLD represents the maximum leakage (tissue) dose after the disc, and is calculated as the maximum value (averaged over a $0.5\;{\text{mm}}^{3}$ volume) for a 23 Gy at R100 therapeutic dose.

### D. Disc position check

Disc misalignments can be checked *a posteriori* using GAFCHROMIC film pasted on the side of the disc exposed to the beam, and, after the treatment, calculating the 50% isodose. Perfect alignment between the disc and the beam axis is shown by a circular shaped dose distribution that is centered with respect to the film border. If misalignment occurs, it is measured by the distance between circular shaped 50% isodose center and film border center.

*In vivo*, disc rotation can be contained. During the target volume thickness measurements the surgeon sometimes inserts, at different points, metallic needles in the tissues as far as the invisible disc face. Thickness variations within 5 mm are usually tolerated. This condition roughly ensures disc rotation of less than 10°.

## III. RESULTS

### A. Monte Carlo validation

(Figure 5a) shows the best match between the experimental (continuous line) and simulated (dash line) PPD for the 10 MeV nominal energy case. These dose values refer to points belonging to the y‐axis at 24 mm depth along the beam x‐axis (R100 PDD reference point). Table 1 summarizes the experimental and simulated reference PPD parameters and the corresponding percentage differences for the four nominal beam energies. The agreement for symmetry and homogeneity was, respectively, within 3% and 5%. Similar results were obtained for 8, 6 and 4 MeV nominal energies.

**Figure 3:**
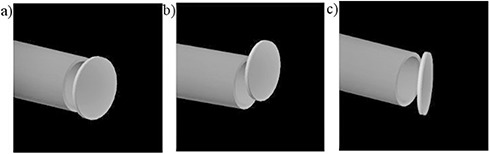
Simulated clinical setup (a): correct disc configuration with shielding disc located at R90, parallel and centered with respect to beam collimation system; erroneous disc configurations (b): shielding disc, at R90, laterally translated with respect to the beam axis; shielding disc (c) initially centered at R90, progressively rotated around an axis tangential to the circular beam aperture (lateral opening).

**Table 1:** Experimental and simulated reference profile parameters.

	*Nominal Energy*	*Field Size (cm)*	*Symmetry (%)*	*Homogeneity (%)*
Exper.		10.26	100.58	101.81
Simul.	10	10.25	102.60	103.79
% Difference		0.1	2.0	1.9
Exper.		10.26	100.40	101.01
Simul.	8	10.23	102.04	102.40
% Difference		0.3	1.6	1.4
Exper.		10.23	100.39	102.00
Simul.	6	10.19	102.12	103.69
% Difference		0.4	1.0	1.7
Exper.		10.10	100.79	101.15
Simul.	4	10.18	102.12	104.52
% Difference		0.8	1.0	3.3

(Figure 5b) shows the best fitting between simulated (continuous lines) and experimental (crosses) PDDs for all available nominal energies (4, 6, 8, and 10 MeV).

As summarized in Table 2, PDD agreement was within 2%. The difference between ${\overset{ˉ}{E}}_{0}$ and $E_{0p}$ can be explained by the large component of scattered electrons produced by the NOVAC7 PMMA collimator system.^(18)^ The electron energy distribution at the water phantom surface is asymmetrically shaped, so that the most probable energy value is considerably higher than the mean energy.

**Table 2:** Experimental and simulated reference PDD values.

	*Nominal Energy*	*R100 (mm)*	*R90 (mm)*	*R50 (mm)*	*R30 (mm)*	$R_{p}$ *(mm)*	${\bar{E}}_{0}$ *(MeV)*	$E_{0p}$ *(MeV)*
Exper.		15.08	24.32	34.80	39.07	45.24	8.11	9.23
Simul.	10	15.01	24.18	34.64	38.94	45.01	8.07	9.18
% Difference		0.5	0.6	0.5	0.3	0.5	0.5	0.5
Exper.		12.18	19.32	28.06	31.75	37.00	6.54	7.58
Simul.	8	12.43	19.69	27.89	31.50	36.62	6.50	7.50
% Difference		2.0	1.9	0.6	0.8	1.0	0.6	1.0
Exper.		8.60	14.09	20.78	23.72	27.92	4.84	5.77
Simul.	6	9.48	14.35	20.83	23.71	27.68	4.85	5.72
% Difference		1.4	1.8	0.2	0.04	0.9	0.2	0.9
Exper.		6.56	10.14	15.18	17.44	20.68	3.54	4.33
Simul.	4	6.51	10.26	15.18	17.45	20.60	3.54	4.31
%Difference		0.8	1.2	0.0	0.06	0.4	0.0	0.5

Table 3 summarizes the initial (at the source) electron beam parameters obtained from the best matching between experimental and simulated PPD and PDD.

**Table 3:** Source electron beam parameters (mean energy. energy, y position, z position, and spread standard deviations).

*Ē (MeV)*	$\sigma_{E}\;(\text{MeV})$	$\sigma_{Y}\;(\text{mm})$	$\sigma_{Z}\;(\text{mm})$	$\sigma_{M}\;(\text{grad})$
10.1	0.5	1.0	1.0	6°
8.3	0.5	1.0	1.0	6°
6.3	0.5	1.0	1.0	6°
4.8	0.5	1.0	1.0	6°

### B. Dose to normal tissue without the disc

In Fig. 6, the zone behind the disc, healthy tissues, is shown as dark areas for all energies, starting at the depth of R90 plus disc thickness. A quantitative analysis of these areas (inset of Fig. 6) reveals that, without the disc, the absorbed dose in the normal tissues would increase almost proportionally with the beam energy, increasing from 7% of the total dose released at 4.8 MeV to 16% at 10.1 MeV.

**Figure 4:**
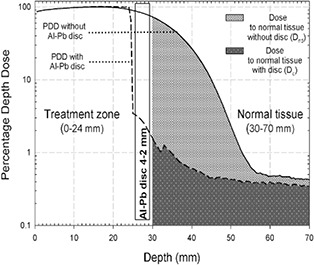
Typical shielding disc effect on normal tissue. Gray and dark gray area values below the PDD curves ($D_{\text{nd}}$ and $D_{d}$) are proportional to the absorbed dose in the normal tissue zone, respectively, without and with the disc. In this case (10 MeV) the shielding factor (SF), defined as $100*(1-D_{d}/D_{\text{nd}})$, is about 96% (i.e., the disc presence almost totally cuts off the dose delivery). (In the vertical axis, the log scale was chosen to better visualize the dark gray area).

### C. Dose distributions analysis: proper disc setup

Figure 7 shows the simulated PDDs, placing the disc at the R90 depths (10.5 mm for 4 MeV, 14.5 mm for 6 MeV, 20.0 mm for 8 MeV, and 24.5 mm for 10 MeV) and at three intermediate positions (12.5 mm for 6 MeV, 16.8 mm for 8 MeV, 22.8 mm for 10 MeV). The inset in Fig. 7 shows the SF and the maximum BSF for each of the above cases.

**Figure 5(a):**
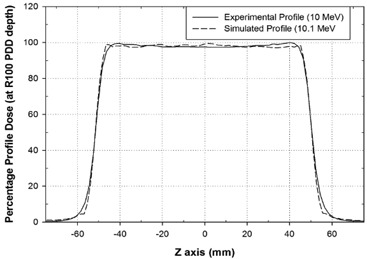
Comparison of experimental and best simulated percentage profile dose (PPD) in reference conditions for the 10 MeV nominal beam energy.

The SF values (from 97.5 to 96.2) show that the presence of the disc reduces the dose to normal tissue, from 2.5% to 3.8% of the total dose released without the disc. Furthermore, the SF slowly decreases with increasing beam energy. The corresponding maximum BSF values vary from 1.1 to 1.0. The mean MLD is 0.23 Gy (1% of the R100 therapeutic dose) (Refer to Fig. 10 below).

**Figure 7:**
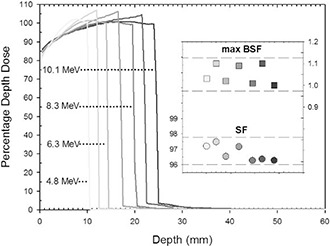
Simulated PDDs with disc positioned at R90 depths (10.5 mm for 4 MeV, 14.5 mm for 6 MeV, 20.0 mm for 8 MeV, and 24.5 mm for 10 MeV) and three intermediate positions (12.5 mm for 6 MeV, 16.8 mm for 8 MeV, 22.8 mm for 10 MeV). Inset shows the corresponding maximum backscattering factor (BSF) and shielding factor (SF) values.

### D. Dose distributions analysis: incorrect disc setup (translations)

Figure 8 shows how the PDD curves change when the disc is translated transversally with respect to the beam axis collimation system (x‐axis). The disc is shifted from 5 mm to 42 mm along y‐axis. This set of simulations refers to 10.1 MeV beam energy with the disc positioned at R90 PDD reference point (24 mm along x‐axis). Similar results were obtained for 8.3, 6.3, and 4.8 MeV (i.e., with the disc placed at depths of 20.0 mm, 14.5 mm, and 10.5 mm, respectively).

**Figure 5(b):**
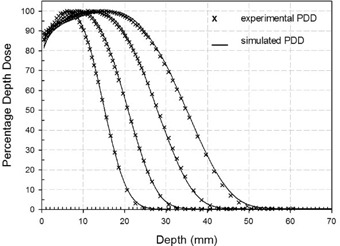
Comparison of experimental and best simulated PDDs in reference conditions for beam energies of 4, 6, 8, 10 MeV.

Shielding performance obviously deteriorates as the disc is shifted away from the beam axis. Due to the low BSF values, the absorbed dose in the treatment region does not change significantly.

These features appear evident in Fig. 9, where the simulated 2D dose distributions in the detector (70 mm depth×150 mm×150 mm surface) at z=0 mm level represent 5, 10, 20, 30, 36, and 42 mm y shift, respectively.

**Figure 6:**
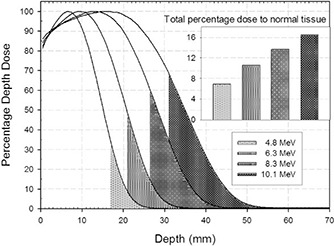
Simulated PDDs for beam energies of 4.8, 6.3, 8.3, and 10.1 MeV. The dark areas refer to the percentage of energy released beyond the disc and outside the treatments zones. The inset quantifies these percentages.

As expected, starting from a 20 mm lateral shift in the disc position (inset 9(c)), a dose leakage into normal tissue occurs through the growing gap between the collimator (100 mmϕ) and the disc borders (120 mm ϕ). This effect is increasingly pronounced for the 30, 36, and 42 mm shifts (insets 9(d), (e) and (f)).

Figure 10 quantifies the above qualitative results for all four beam energies. The upper part shows the calculated SF value versus the disc translation. Within 5 mm displacement, the SF varies from 97.3 to 94.0. From 5 mm to 14 mm, the SF decreases and its value is almost within the 90 value. From 14 mm to 42 mm, it decreases linearly down to 60. The percent SF spread, due to different energy beams, varies from 3.0% at 5 mm to 4.4% at 42 mm. The lower part of Fig. 10 shows MLD versus disc translation. The MLD is calculated, averaged on the four beam energies, and varies from 0.69 Gy at 5 mm to 16.79 Gy values at 42 mm.

### E. Dose distributions analysis: incorrect disc setup (rotations)

In this simulations' set, we considered the disc positioned always at R90 PDD reference point but rotated around its diameter parallel to the z‐axis. As for disc translations, we have simulated 2D dose distributions in the detector (70 mm depth×150 mm×150 mm surface) at z=0 mm level.

Figure 11 shows these 2D dose distributions with the disc for the 10.1 MeV beam energy case, progressively rotated at 5.0°, 10.0°, 15.0°, 20.0°, 25.0°, and 30.0° (from insets (a) to (f), respectively).

**Figure 8:**
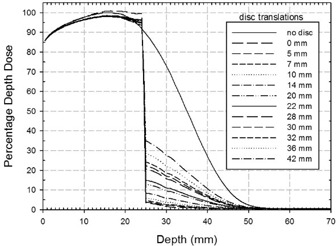
Simulated PDDs with the disc positioned at depth of 24 mm (case 10.1 MeV), and translated from 0 mm to 42 mm transversally with respect to the axis of the beam collimation system.

For rotation angles up 20° (inset (d)), no energy deposition behind the disc is evident. For higher angle values the shielding performance deteriorates, as is evident in the left lower zones in insets (e) and (f). Figure 12 shows the isodose for the 30° case (inset (f)). In the left lower region behind the disc, a 10% portion of the maximum dose (23 Gy) is distributed roughly over a $2\times 1{\;\text{cm}}^{2}$ surface.

**Figure 9:**
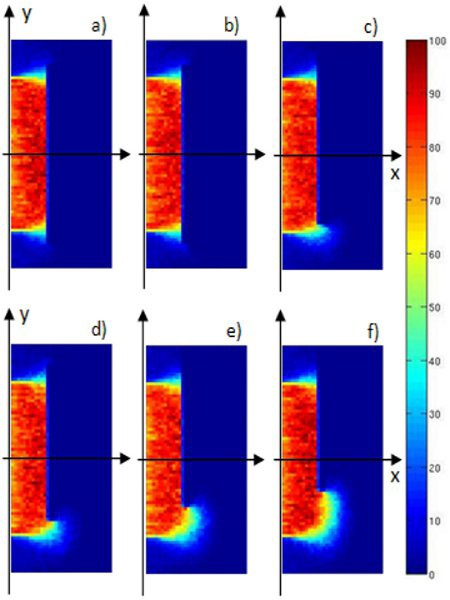
Simulated 2D dose distributions inside the detector (70 mm along x×150 mm along y×150 mm along z) in the x‐y plane at z=0 (central plane) when the disc is positioned at x=24 mm (case 10.1 MeV), and translated +5 (a), +10 (b), +20 (c), +30 (d), +36 (e), and +42 (f) mm along y (i.e., transversally with respect to the axis of the beam collimation system (x‐axis)). In each inset, the beam enters the detector on the left side and the shielding disc is on the right.

For rotation angles higher than 10° (from inset (c) to (f)), the dose distribution homogeneity is also greatly affected and a more pronounced low‐dose region appears. Figure 13 shows the corresponding dose–volume histograms. It is evident as the underdosed region (less than 80% of the maximum dose released) increases from 5% of the total area at 10° up to a 20% at 30°.

**Figure 10:**
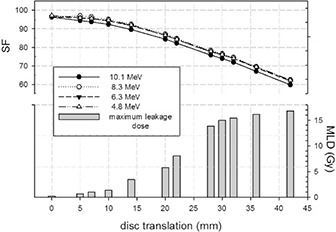
Shielding factor (SF) (upper part) calculated with disc positioned at R90 (case 10.1, 8.3, 6.3, and 4.8 MeV) and translated from 0 mm to 42 mm along y‐axis perpendicularly with respect to the axis of the beam collimation system. The lower part of the the corresponding maximum leakage dose (MLD) in the normal tissue zone. The MLD is averaged on beam energies.

Figure 14 shows the dose distribution (at 10.1 MeV beam energy) when the disc is tilted 180° (i.e., with the lead face in front of the beam). A BSF of 27.8 at R90 is not considered compatible with a clinically acceptable treatment.

**Figure 11:**
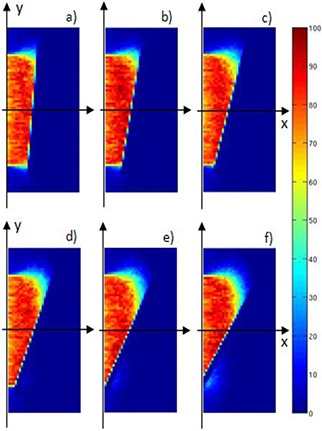
Simulated 2D dose distributions inside the detector (70 mm along x×150 mm along y×150 mm along z) in the x‐y plane at z=0 (central plane) with disc at x=24 mm (case 10.1 MeV), and rotated 5.0° (a), 10.0° (b), 15.0° (c), 20.0° (d), 25.0° (d), and 30.0° (f) around its diameter parallel to the z‐axis.

**Figure 13:**
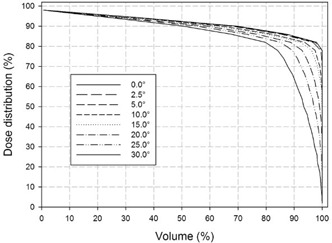
Dose‐volume curves relative to disc positioned at x=24 mm (case 10.1 MeV) and rotated from 2.5° to 30° along z‐axis perpendicularly to the beam axis. For 30° tilt angle, 20% of the treatment volume is underdosed (less than 80% of the maximum dose).

**Figure 14:**
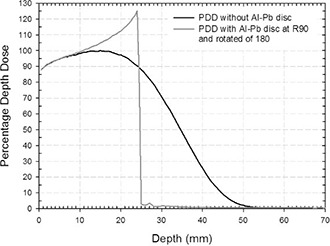
Simulated PDDs with the Al‐Pb disc 180° tilted with the lead face in front of the beam and positioned at R90 (dark grey curve). The backscattering factor (BSF) is not considered compatible with a clinically acceptable treatment.

### F. Disc position check

Figure 15 (left) shows a typical clinical GAFCHROMIC film for an 80 mm disc diameter. The drawn internal circle represents the 50% isodose. The distance between this internal circle center and film border center corresponds to a disc misalignment of 4 mm. The reported variability in disc rotation angle for this case was within 10°. Figure 15 (right) is the corresponding simulated dose distribution, where the experimental 50% isodose (black dotted circle) adequately superimposes the simulated ones (clear blue zone).

**Figure 12:**
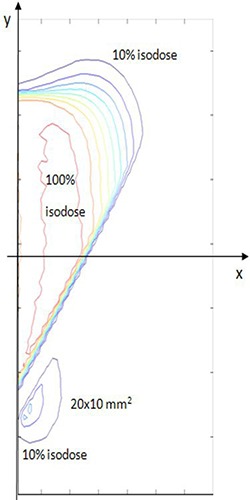
Contour dose distribution relative to inset (f) of the disc positioned at x=24 mm (case 10.1 MeV), with a lateral opening of 30.0°. An approximately $2\times 1{\;\text{cm}}^{2}$ surface 10%–20% dosed appears in the left lower part behind the shielding disc.

**Figure 15:**
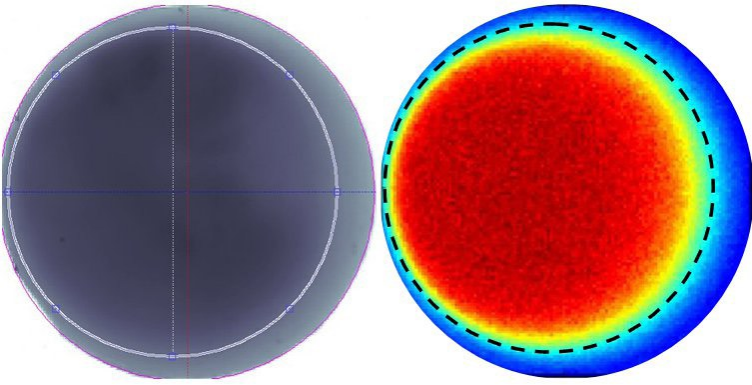
(left) Typical clinical GAFCHROMIC film for a 80 mm disc diameter. The drawn internal circle represents the 50% isodose. The distance between this internal circle center and film border center corresponds to a disc misalignment of 4 mm. (right) Corresponding simulated dose distribution. The experimental 50% isodose (black dotted circle) adequately superimposes the simulated ones (clear blue zone).

## IV. DISCUSSION

In breast IOERT treatment, the unavailability of a Monte Carlo TPS for accurate dose distribution studies and a controlled and automated delivering dose system makes the protection of internal normal tissue a crucial point: shielding‐disc misalignment is very relevant and wrong orientation risks could occur. Consequently, the treatment zone could receive an erroneous or nonuniform dose delivery, and normal tissues could be exposed to radiation.

It is evident from Fig. 6 that, without using a shielding disc, the dose to normal tissues grows linearly with the beam energy, reaching 16% of the total dose released. This confirms the use of a shielding disc as a first benchmark in the clinical setup of high‐dose IOERT treatment. Fortunately today such use is well‐established practice.^(6)^

Another major concern regards disc positioning. As expected, disc alignment and centering with respect to the applicator is the most correct setup configuration. In the case of the Al‐Pb 4–2 mm disc, the BSF is no higher than 10%, the mean shielding is 97%, and the mean residual dose in the healthy tissue zone is less than 1% for all considered beam energies and disc positions. Similar results were obtained for other discs of different materials and thicknesses.^8^, ^9^

A further point is that, nowadays, the effects on dose distribution of disc misalignment and/or rotation can be quantified. Lateral translation equal to or greater than the difference between the shielding disc radius and the internal radius of the applicator must be avoided. When the disc is translated with respect to its correct position, it does not significantly influence the dose delivery to the treatment zone. However, such translation does compromise the shielding of normal tissues. In our case (disc radius 60 mm and applicator internal radius 50 mm), 5 mm to 10 mm translation already led to hot spots of 1–2 Gy dose leakage. For a displacement of about 20 mm, the hot spots could reach 6 Gy. Thus the gap between the collimator and the disc borders must be firmly checked.

With disc rotations of up to 10°, the dose distribution homogeneity in the treatment zone remains acceptable, varying only 2% with respect to the correct situation (0°), as is the shielding of the normal tissues. However above 20°, increasing areas of normal tissue become irradiated, the dose ranging from 2.3 to 4.6 Gy. Wrong disc orientation (180°) is prohibitive. Figure 14 shows such a case, the dark grey curve showing a BSF of 27.8, which definitely does not guarantee a reasonably uniform dose distribution within the target. Such a condition makes it difficult to ensure radiotherapy treatment efficacy. This high BSF value originates from the electron backscattering effect, which is linearly proportional to the atomic number of the disc material in front of the beam.

Finally, the validity of the simulation model and the above results are quite evident on comparing the dose distributions measured by the GAFCHROMICS with those calculated by the Monte Carlo method (Fig. 15).

## V. CONCLUSIONS

The simulations confirm how, in general, misalignment of the shielding disc results in a greater risk condition than wrong orientation.^(11)^ Information obtained by checking the disc position makes it possible to estimate, though only *a posteriori*, the dose distribution in the target and normal tissue. A tolerance range for disc misalignment and rotation has now been delineated, and such information will help the surgical team choose the most correct clinical setup, and allow the quantification, *a posteriori*, of the degree of success or failure of an IOERT breast treatment. Our next step is to project an automatic device capable of alerting surgeons, in real time, when the disc is outside the accepted tolerance position, thus allowing adjustments to be made to its positioning. We believe that this feature would contribute to increasing IOERT intrinsic treatment quality and patient safety.

## ACKNOWLEDGMENTS

The authors are grateful for the kind assistance and support given by New Radiant Technology NRT S.P.A., and would like to thank Salvatore Barone for his collaboration. The authors would also like to thank Pablo Cirrone and Francesco Romano, researchers at Laboratori Nazionali del Sud (LNS‐INFN), for useful discussions.
